# Supplementary material for: Structural and Functional Studies of S-(2-Carboxyethyl)-L-Cysteine and S-(2-Carboxyethyl)-l-Cysteine Sulfoxide
Source: Molecules. 2022 Aug 20;27(16):5317. doi: 10.3390/molecules27165317 (PMC9414067; doi:10.3390/molecules27165317)
Supplement: Supplementary file 1 [file molecules-27-05317-s001.zip › molecules-1878636-supplementary.pdf]

Supplementary Materials to

Structural and functional studies of *S*-(2-carboxyethyl)-L-cysteine and *S*-(2-carboxyethyl)-L-cysteine sulfoxide

James K. Waters, Valeri V. Mossine,* Steven P. Kelley and Thomas P. Mawhinney

Table S1. Crystal data, data collection, and structure refinement details

Molecule	**1**, β-CEC	**2**, (4*R*)-β-CECO	**3**, (4*S*)-β-CECO
Crystal data			
Chemical formula	$C_6H_{11}NO_4S$	$C_6H_{11}NO_5S$	$C_6H_{11}NO_5S$
M_r	193.22	209.22	209.22
Crystal system, space group	Orthorhombic, $P2_12_12_1$	Orthorhombic, $P2_12_12_1$	Monoclinic, $P2_1$
Temperature (K)	100	150	295
a, *b*, *c* (Å)	5.0291 (17), 6.983 (2), 22.816 (8)	5.1735 (5), 8.6999 (8), 18.2013 (16)	4.9127 (1), 18.4673 (4), 5.1750 (1)
α, β, γ (°)	90, 90, 90	90, 90, 90	90, 112.4071 (8), 90
V (Å^3)	801.2 (5)	819.22 (13)	434.05 (2)
Z	4	4	2
Radiation type	Mo *K*α	Mo *K*α	Cu *K*α
μ (mm^{-1})	0.384	0.384	3.32
Crystal size (mm)	0.25 × 0.15 × 0.02	0.5 × 0.2 × 0.1	0.25 × 0.21 × 0.14
Data collection			
Diffractometer	Bruker *APEX* II area detector	Bruker *APEX* II area detector	Bruker *APEX* II area detector
Absorption correction	Multi-scan Bruker AXScale	Multi-scan Bruker AXScale	Multi-scan Bruker AXScale
T_{min}, T_{max}	0.637, 0.745	0.696, 0.747	0.638, 0.754
No. of measured, independent and observed [$I > 2\sigma(I)$] reflections	10893, 1638, 1418	34600, 3139, 2991	5933, 1689, 1687
R_{int}	0.072	0.035	0.022
θ_{max}, θ_{min} (°)	26.4, 1.8	33.3, 2.2	73.9, 4.8
Refinement			
$R[F^2 > 2\sigma(F^2)]$, $wR(F^2)$, S	0.038, 0.092, 0.98	0.029, 0.079, 1.09	0.031, 0.083, 1.06
No. of reflections	1638	3139	1689
No. of parameters	142	130	131

No. of restraints	0	0	4
H-atom treatment	Only H-atom coordinates refined	H atoms treated by a mixture of independent and constrained refinement	H atoms treated by a mixture of independent and constrained refinement
$\Delta\rangle_{max}$, $\Delta\rangle_{min}$ (e Å^{-3})	0.52, -0.24	0.64, -0.23	0.22, -0.25
Absolute structure	Flack x determined using 514 quotients [(I+)-(I-)]/[(I+)+(I-)] (Parsons, Flack and Wagner, Acta Cryst. B69 (2013) 249-259).	Flack x determined using 1202 quotients [(I+)-(I-)]/[(I+)+(I-)]	Flack x determined using 785 quotients [(I+)-(I-)]/[(I+)+(I-)]
Absolute structure parameter	-0.04 (7)	0.005 (17)	-0.021 (19)

Special details

Geometry. All esds (except the esd in the dihedral angle between two l.s. planes) are estimated using the full covariance matrix. The cell esds are taken into account individually in the estimation of esds in distances, angles and torsion angles; correlations between esds in cell parameters are only used when they are defined by crystal symmetry. An approximate (isotropic) treatment of cell esds is used for estimating esds involving l.s. planes.

Table S2. Fractional atomic coordinates and isotropic or equivalent isotropic displacement parameters (Å^2) for S-(2-carboxyethyl)-L-cysteine (β-CEC)

	x	*y*	*z*	U_{iso}*/U_{eq}
S1	0.43978 (18)	0.46361 (14)	0.90369 (4)	0.0163 (3)
O1	1.1681 (4)	0.5566 (5)	0.76059 (10)	0.0170 (6)
O4	0.5097 (5)	0.4544 (4)	1.12166 (11)	0.0174 (6)
O2	0.7656 (5)	0.5714 (4)	0.71995 (10)	0.0169 (6)
O3	0.8816 (4)	0.5617 (4)	1.07883 (10)	0.0177 (6)
C1	0.9229 (7)	0.5839 (5)	0.76202 (14)	0.0119 (7)
N1	0.5862 (6)	0.7757 (5)	0.81330 (13)	0.0116 (6)
C4	0.6462 (7)	0.5319 (6)	0.96536 (15)	0.0157 (7)
H4A	0.832 (8)	0.482 (6)	0.9594 (16)	0.019*
H4B	0.646 (8)	0.675 (7)	0.9692 (17)	0.019*
C5	0.5161 (7)	0.4526 (6)	1.01979 (14)	0.0161 (8)
H5A	0.332 (8)	0.495 (6)	1.0225 (16)	0.019*
H5B	0.515 (8)	0.308 (6)	1.0187 (16)	0.019*

C6	0.6585 (7)	0.4966 (5)	1.07614 (15)	0.0141 (8)
C3	0.6763 (7)	0.4416 (6)	0.84455 (14)	0.0152 (8)
H3A	0.813 (8)	0.363 (6)	0.8542 (18)	0.018*
H3B	0.566 (8)	0.380 (6)	0.8145 (17)	0.018*
C2	0.7950 (7)	0.6278 (6)	0.82149 (15)	0.0126 (8)
H2	0.931 (8)	0.676 (6)	0.8469 (16)	0.015*
H1A	0.530 (8)	0.805 (6)	0.8469 (18)	0.019*
H1B	0.461 (9)	0.732 (6)	0.7946 (17)	0.019*
H1C	0.647 (8)	0.871 (6)	0.7929 (18)	0.019*
H4	0.584 (8)	0.452 (6)	1.1526 (17)	0.019*

Table S3. Atomic displacement parameters (Å^2) for S-(2-carboxyethyl)-L-cysteine (β-CEC)

	U^{11}	U^{22}	U^{33}	U^{12}	U^{13}	U^{23}
S1	0.0157 (4)	0.0208 (5)	0.0124 (4)	-0.0041 (4)	0.0003 (3)	0.0002 (4)
O1	0.0091 (11)	0.0257 (17)	0.0161 (12)	0.0011 (13)	0.0004 (9)	-0.0040 (12)
O4	0.0152 (13)	0.0267 (15)	0.0104 (11)	-0.0015 (12)	-0.0008 (9)	0.0021 (12)
O2	0.0111 (11)	0.0271 (17)	0.0126 (12)	-0.0017 (12)	-0.0004 (9)	-0.0037 (11)
O3	0.0154 (13)	0.0211 (15)	0.0166 (12)	-0.0057 (12)	-0.0001 (9)	-0.0016 (11)
C1	0.0108 (15)	0.0111 (19)	0.0139 (16)	-0.0002 (16)	0.0015 (13)	-0.0021 (13)
N1	0.0104 (15)	0.0152 (17)	0.0091 (14)	0.0002 (14)	0.0009 (12)	0.0011 (12)
C4	0.0166 (16)	0.016 (2)	0.0148 (16)	-0.0021 (16)	-0.0035 (13)	0.0001 (16)
C5	0.0151 (18)	0.020 (2)	0.0134 (17)	-0.0020 (18)	-0.0011 (12)	-0.0004 (15)
C6	0.0160 (15)	0.011 (2)	0.0154 (15)	0.0025 (14)	-0.0002 (13)	0.0001 (13)
C3	0.0184 (17)	0.018 (2)	0.0096 (16)	0.0013 (18)	0.0000 (13)	-0.0011 (16)
C2	0.0090 (16)	0.017 (2)	0.0119 (16)	0.0016 (14)	-0.0012 (13)	-0.0013 (14)

Table S4. Geometric parameters (Å, º) for S-(2-carboxyethyl)-L-cysteine (β-CEC)

S1—C4	1.812 (4)	C1—C2	1.533 (5)
S1—C3	1.805 (4)	N1—C2	1.485 (5)
O1—C1	1.248 (4)	C4—C5	1.509 (5)
O4—C6	1.313 (4)	C5—C6	1.504 (5)
O2—C1	1.247 (4)	C3—C2	1.525 (6)
O3—C6	1.212 (4)	O4—H4	0.80 (4)

N1—H1A	0.84 (4)	N1—H1B	0.82 (4)
N1—H1C	0.87 (4)	C2—H2	0.96 (4)
C3—H3A	0.91 (4)	C3—H3B	0.98 (4)
C4—H4A	1.01 (4)	C4—H4B	1.00 (5)
C5—H5A	0.97 (4)	C5—H5B	1.01 (4)
C3—S1—C4	103.01 (17)	O3—C6—O4	124.8 (3)
O1—C1—C2	117.9 (3)	O3—C6—C5	124.1 (3)
O2—C1—O1	126.6 (3)	C2—C3—S1	116.3 (3)
O2—C1—C2	115.4 (3)	N1—C2—C1	108.9 (3)
C5—C4—S1	107.1 (3)	N1—C2—C3	111.1 (3)
C6—C5—C4	115.0 (3)	C3—C2—C1	107.4 (3)
O4—C6—C5	111.0 (3)	C6—O4—H4	116 (3)
C2—N1—H1A	107 (3)	C2—N1—H1B	111 (3)
C2—N1—H1C	111 (3)	H1A—N1—H1B	108 (4)
H1A—N1—H1C	115 (4)	H1B—N1—H1C	106 (4)
N1—C2—H2	110 (2)	C1—C2—H2	108 (2)
C3—C2—H2	112 (2)	S1—C3—H3A	112 (3)
S1—C3—H3B	101 (2)	C2—C3—H3A	108 (3)
C2—C3—H3B	11 1(2)	H3A—C3—H3B	109 (4)
S1—C4—H4A	110 (2)	S1—C4—H4B	109 (2)
C5—C4—H4A	113 (2)	C5—C4—H4B	107 (2)
H4A—C4—H4B	111 (3)	C4—C5—H5A	111 (2)
C4—C5—H5B	110 (2)	C6—C5—H5A	110 (2)
C6—C5—H5B	103 (2)	H5A—C5—H5B	108 (3)
S1—C4—C5—C6	-179.4 (3)	O2—C1—C2—C3	78.6 (4)
S1—C3—C2—C1	-164.1 (2)	C4—S1—C3—C2	-71.3 (3)
S1—C3—C2—N1	-45.1 (4)	C4—C5—C6—O4	168.3 (3)
O1—C1—C2—N1	141.0 (3)	C4—C5—C6—O3	-11.9 (6)
O1—C1—C2—C3	-98.5 (4)	C3—S1—C4—C5	-149.6 (3)
O2—C1—C2—N1	-41.8 (4)	C4—S1—C3—H3A	53 (3)
C4—S1—C3—H3B	169 (3)	C3—S1—C4—H4A	-27 (3)
C3—S1—C4—H4B	95 (2)	H4—O4—C6—O3	-13 (3)
O2—C1—C2—H2	-161 (3)	C1—C2—N1—H1A	-175 (3)
C1—C2—N1—H1B	68 (3)	C1—C2—N1—H1C	-49 (3)
C3—C2—N1—H1A	67 (3)	C3—C2—N1—H1B	-50 (3)

C3—C2—N1—H1C	-167 (3)	H2—C2—N1—H1A	-57 (4)
H2—C2—N1—H1B	-174 (4)	H2—C2—N1—H1C	69 (4)
N1—C2—C3—H3A	-171 (3)	N1—C2—C3—H3B	69 (3)
C1—C2—C3—H3A	70 (3)	C1—C2—C3—H3B	-50 (3)
H2—C2—C3—S1	78 (3)	H2—C2—C3—H3A	-48 (4)
H2—C2—C3—H3B	-168 (4)	S1—C4—C5—H5A	-55 (3)
S1—C4—C5—H5B	64 (2)	H4A—C4—C5—C6	60 (3)
H4A—C4—C5—H5A	-175 (4)	H4A—C4—C5—H5B	-56 (3)
H4B—C4—C5—C6	-62 (2)	H4B—C4—C5—H5A	63 (3)
H4B—C4—C5—H5B	-179 (3)	H5A—C5—C6—O3	-137 (3)
H5A—C5—C6—O4	43 (3)	H5B—C5—C6—O3	108 (2)
H5B—C5—C6—O4	-71 (2)		

Table S5. Hydrogen-bond geometry (Å, º) for S-(2-carboxyethyl)-L-cysteine (β-CEC)

D—H···*A*	*D*—H	H···*A*	*D*···*A*	*D*—H···*A*
C4—H4*B*···O3^{i}	1.00 (5)	2.52 (4)	3.293 (5)	134 (3)
C5—H5*A*···O3^{ii}	0.98 (4)	2.64 (4)	3.546 (4)	154 (3)
C3—H3*A*···O4^{iii}	0.90 (4)	2.49 (4)	3.324 (5)	153 (4)
C3—H3*B*···O1^{ii}	0.98 (4)	2.65 (4)	3.293 (4)	123 (3)
N1—H1*A*···S1	0.84 (4)	2.75 (4)	3.090 (4)	106 (3)
N1—H1*A*···O3^{i}	0.84 (4)	2.07 (4)	2.899 (4)	167 (4)
N1—H1*B*···O1^{ii}	0.82 (4)	2.07 (4)	2.865 (4)	164 (4)
N1—H1*C*···O1^{iv}	0.87 (5)	2.01 (5)	2.866 (4)	169 (4)
N1—H1*C*···O2^{v}	0.87 (5)	2.52(4)	2.823(4)	101(3)
O4—H4···O2^{vi}	0.80 (4)	1.72 (4)	2.518 (3)	175 (5)

Symmetry codes: (i) *x*-1/2, -*y*+3/2, -*z*+2; (ii) *x*-1, *y*, *z*; (iii) *x*+1/2, -*y*+1/2, -*z*+2; (iv) -*x*+2, *y*+1/2, -*z*+3/2; (v) –*x*+1, *y*+1/2, -*z*+3/2; (vi) -*x*+3/2, -*y*+1, *z*+1/2.

Table S6. Fractional atomic coordinates and isotropic or equivalent isotropic displacement parameters (Å^2) for (4R)-S-(2-carboxyethyl)-L-cysteine sulfoxide (4R-β-CECO)

	x	*y*	*z*	U_{iso}*/U_{eq}
S1	0.73829 (7)	0.32965 (4)	0.85377 (2)	0.01216 (9)
O3	1.0264 (2)	0.30851 (15)	0.85157 (7)	0.0179 (2)

O1	0.3118 (2)	0.17495 (14)	0.71959 (7)	0.0151 (2)
O2	0.7223 (2)	0.18241 (17)	0.68196 (7)	0.0217 (3)
N1	0.8958 (3)	-0.00913 (15)	0.78166 (8)	0.0114 (2)
C6	0.7432 (3)	0.60253 (16)	1.03630 (8)	0.0123 (3)
O4	0.5662 (3)	0.55071 (17)	1.07266 (7)	0.0195 (3)
O5	0.8935 (3)	0.71329 (17)	1.05769 (7)	0.0211 (3)
C1	0.5430 (3)	0.14241 (17)	0.72471 (8)	0.0111 (3)
C2	0.6277 (3)	0.04813 (18)	0.79186 (8)	0.0100 (2)
H2	0.511061	-0.043229	0.795487	0.012*
C5	0.8151 (3)	0.53983 (19)	0.96189 (8)	0.0135 (3)
H5A	1.001262	0.513613	0.961281	0.016*
H5B	0.784214	0.619356	0.923986	0.016*
C3	0.5966 (3)	0.14184 (17)	0.86235 (8)	0.0117 (2)
H3A	0.679968	0.086476	0.903542	0.014*
H3B	0.410462	0.152447	0.873910	0.014*
C4	0.6570 (3)	0.3975 (2)	0.94402 (9)	0.0159 (3)
H4A	0.470424	0.422433	0.946212	0.019*
H4B	0.692857	0.316141	0.980658	0.019*
H1A	0.888 (6)	-0.080 (3)	0.7439 (14)	0.024*
H1B	0.978 (6)	0.068 (3)	0.7677 (15)	0.024*
H1C	0.950 (6)	-0.052 (3)	0.8235 (15)	0.024*
H5	0.842 (6)	0.745 (3)	1.1005 (15)	0.024*

Table S7. Atomic displacement parameters (Å^2) for (4R)-S-(2-carboxyethyl)-L-cysteine sulfoxide (4R-β-CECO)

	U^{11}	U^{22}	U^{33}	U^{12}	U^{13}	U^{23}
S1	0.01536 (15)	0.01030 (15)	0.01083 (15)	-0.00017 (13)	0.00156 (13)	-0.00065 (11)
O3	0.0137 (5)	0.0185 (6)	0.0215 (6)	-0.0006 (4)	0.0052 (4)	-0.0022 (5)
O1	0.0124 (5)	0.0156 (5)	0.0173 (5)	0.0027 (4)	-0.0011 (4)	0.0020 (4)
O2	0.0145 (5)	0.0328 (7)	0.0177 (5)	0.0025 (6)	0.0023 (4)	0.0133 (5)
N1	0.0109 (5)	0.0120 (5)	0.0112 (5)	0.0015 (4)	-0.0003 (4)	0.0010 (4)
C6	0.0145 (6)	0.0121 (6)	0.0103 (6)	0.0017 (6)	-0.0004 (5)	-0.0003 (4)
O4	0.0237 (6)	0.0209 (6)	0.0139 (5)	-0.0065 (5)	0.0060 (5)	-0.0022 (4)
O5	0.0232 (6)	0.0242 (6)	0.0158 (6)	-0.0098 (5)	0.0059 (5)	-0.0085 (5)

C1	0.0133 (6)	0.0096 (6)	0.0103 (6)	-0.0006 (4)	-0.0007 (5)	0.0007 (5)
C2	0.0096 (6)	0.0111 (6)	0.0094 (6)	0.0005 (5)	0.0000 (4)	0.0005 (5)
C5	0.0145 (6)	0.0135 (6)	0.0124 (6)	-0.0009 (5)	0.0022 (5)	-0.0028 (5)
C3	0.0132 (6)	0.0112 (6)	0.0107 (6)	-0.0006 (5)	0.0016 (5)	-0.0004 (5)
C4	0.0178 (7)	0.0164 (7)	0.0135 (6)	-0.0033 (6)	0.0047 (5)	-0.0044 (5)

Table S8. Geometric parameters (Å, º) for (4R)-S-(2-carboxyethyl)-L-cysteine sulfoxide (4R-β-CECO)

S1—O3	1.5026 (12)	C6—O4	1.216 (2)
S1—C3	1.7977 (15)	C6—O5	1.298 (2)
S1—C4	1.7954 (16)	C6—C5	1.507 (2)
O1—C1	1.2328 (18)	C1—C2	1.536 (2)
O2—C1	1.2595 (19)	C2—C3	1.529 (2)
N1—C2	1.486 (2)	C5—C4	1.519 (2)
O5—H5	0.87 (3)	N1—H1A	0.92 (3)
N1—H1B	0.83 (3)	N1—H1C	0.89 (3)
C2—H2	1.00	C3—H3A	0.99
C3—H3B	0.99	C4—H4A	0.99
C4—H4B	0.99	C5—H5A	0.99
C5—H5B	0.99		
O3—S1—C3	107.19 (7)	O2—C1—C2	115.42 (13)
O3—S1—C4	107.28 (8)	N1—C2—C1	110.24 (12)
C4—S1—C3	97.11 (7)	N1—C2—C3	112.45 (12)
O4—C6—O5	124.26 (14)	C3—C2—C1	110.68 (12)
O4—C6—C5	122.73 (14)	C6—C5—C4	110.78 (13)
O5—C6—C5	112.99 (14)	C2—C3—S1	111.65 (10)
O1—C1—O2	127.19 (15)	C5—C4—S1	109.74 (11)
O1—C1—C2	117.35 (13)	C6—O5—H5	108.7 (19)
C2—N1—H1A	106.0 (19)	C2—N1—H1B	104 (2)
C2—N1—H1C	109.1 (19)	H1A—N1—H1B	109 (3)
H1A—N1—H1C	112 (2)	H1B—N1—H1C	116 (3)
N1—C2—H2	108	C1—C2—H2	108
C3—C2—H2	108	S1—C3—H3A	109
S1—C3—H3B	109	C2—C3—H3A	109

C2—C3—H3B	109	H3A—C3—H3B	108
S1—C4—H4A	110	S1—C4—H4B	110
C5—C4—H4A	110	C5—C4—H4B	110
H4A—C4—H4B	108	C4—C5—H5A	109
C4—C5—H5B	109	C6—C5—H5A	109
C6—C5—H5B	109	H5A—C5—H5B	108
O3—S1—C3—C2	-70.17 (12)	C4—S1—C3—C2	179.22 (11)
O3—S1—C4—C5	53.95 (13)	C3—S1—C4—C5	164.48 (11)
O1—C1—C2—N1	167.85 (13)	O1—C1—C2—C3	-67.09 (18)
O2—C1—C2—N1	-14.48 (19)	O2—C1—C2—C3	110.58 (16)
N1—C2—C3—S1	74.62 (14)	C1—C2—C3—S1	-49.18 (15)
S1—C4—C5—C6	177.88 (10)	C4—C5—C6—O4	-5.7 (2)
C4—C5—C6—O5	172.47 (14)	O3—S1—C3—H3A	51
O3—S1—C3—H3B	169	C4—S1—C3—H3A	-60
C4—S1—C3—H3B	58	O3—S1—C4—H4A	175
O3—S1—C4—H4B	-67	C3—S1—C4—H4A	-75
C3—S1—C4—H4B	44	H5—O5—C6—O4	-2 (2)
H5—O5—C6—C5	180 (2)	H1A—N1—C2—C1	-67.6 (17)
H1A—N1—C2—C3	168.4 (17)	H1A—N1—C2—H2	50
H1B—N1—C2—C1	48 (2)	H1B—N1—C2—C3	-76 (2)
H1B—N1—C2—H2	165	H1C—N1—C2—C1	172.0 (18)
H1C—N1—C2—C3	48.0 (18)	H1C—N1—C2—H2	-71
O1—C1—C2—H2	50	O2—C1—C2—H2	-132
N1—C2—C3—H3A	-46	N1—C2—C3—H3B	-164
C1—C2—C3—H3A	-170	C1—C2—C3—H3B	72
H2—C2—C3—S1	-167	H2—C2—C3—H3A	72
H2—C2—C3—H3B	-46	S1—C4—C5—H5A	-61
S1—C4—C5—H5B	57	H4A—C4—C5—C6	57
H4A—C4—C5—H5A	178	H4A—C4—C5—H5B	-64
H4B—C4—C5—C6	-61	H4B—C4—C5—H5A	59
H4B—C4—C5—H5B	178	H5A—C5—C6—O4	-127
H5A—C5—C6—O5	52	H5B—C5—C6—O4	115
H5B—C5—C6—O5	-67		

Table S9. Hydrogen-bond geometry (Å, º) for (4R)-S-(2-carboxyethyl)-L-cysteine sulfoxide (4R-

β-CECO)

D—H···*A*	*D*—H	H···*A*	*D*···*A*	*D*—H···*A*
O5—H5···O2^{i}	0.87 (3)	1.65 (3)	2.5097 (19)	173 (3)
N1—H1*A*···S1^{ii}	0.92 (3)	2.74 (3)	3.4099 (15)	130 (2)
N1—H1*A*···O1^{iii}	0.92 (3)	2.46 (3)	2.9510 (18)	113 (2)
N1—H1*A*···O3^{ii}	0.92 (3)	2.04 (3)	2.9257 (19)	160 (3)
N1—H1*B*···O1^{iv}	0.83 (3)	2.15 (3)	2.9108 (19)	152 (3)
N1—H1*B*···O2	0.83 (3)	2.28(3)	2.622(2)	105 (2)
N1—H1*C*···O4^{v}	0.91 (3)	1.97 (3)	2.815 (2)	155 (2)
C3—H3*A*···O4^{v}	0.99	2.37	3.179 (2)	139
C3—H3*B*···O3^{vi}	0.99	2.44	3.2929 (19)	144
C5—H5*B* ···O5^{vii}	0.99	2.51	3.082 (2)	116

Symmetry codes: (i) 3/2-x,1-y,1/2+z; (ii) 2-x,-1/2+y,3/2-z; (iii) 1-x,-1/2+y,3/2-z; (iv) 1+x,y,z; (v) 1/2+x,1/2-y,2-z; (vi) -1+x,y,z; (vii) -1/2+x,3/2-y,2-z.

Table S10. Fractional atomic coordinates and isotropic or equivalent isotropic displacement parameters (Å^2) for (4S)-S-(2-carboxyethyl)-L-cysteine sulfoxide (4S-β-CECO)

	x	*y*	*z*	U_{iso}*/U_{eq}
S1	0.91875 (14)	0.79505 (4)	1.07001 (12)	0.0273 (2)
O3	0.6140 (5)	0.79884 (16)	1.0696 (5)	0.0422 (5)
C6	0.7692 (7)	0.63116 (16)	0.4878 (7)	0.0284 (6)
O5	0.5384 (5)	0.60142 (14)	0.2916 (5)	0.0380 (6)
C3	0.9215 (7)	0.85487 (16)	0.7937 (6)	0.0273 (6)
H3A	1.107084	0.849471	0.769983	0.033*
H3B	0.764959	0.840832	0.619793	0.033*
C4	0.9551 (7)	0.71126 (17)	0.9038 (7)	0.0324 (7)
H4A	1.120493	0.715993	0.846258	0.039*
H4B	1.002211	0.672830	1.041785	0.039*
O4	1.0178 (5)	0.61453 (15)	0.5262 (7)	0.0506 (8)
C2	0.8808 (5)	0.93430 (15)	0.8544 (5)	0.0204 (5)
H2	0.715186	0.938393	0.914966	0.025*
C5	0.6884 (7)	0.68850 (17)	0.6528 (7)	0.0323 (6)
H5A	0.538610	0.669837	0.714512	0.039*
H5B	0.607466	0.730223	0.534534	0.039*

O2	1.3214 (5)	1.00516 (15)	1.0153 (5)	0.0388 (6)
O1	1.2056 (5)	0.94357 (12)	1.3257 (4)	0.0306 (5)
N1	0.8109 (5)	0.97479 (15)	0.5890 (5)	0.0250 (5)
C1	1.1582 (5)	0.96473 (15)	1.0824 (5)	0.0224 (5)
H1A	0.836 (9)	1.0208 (16)	0.634 (8)	0.034*
H5	0.591 (9)	0.577 (3)	0.216 (10)	0.034*
H1B	0.922 (8)	0.961 (2)	0.496 (8)	0.034*
H1C	0.624 (6)	0.972 (2)	0.469 (7)	0.034*

Table S11. Atomic displacement parameters (Å^2) for (4S)-S-(2-carboxyethyl)-L-cysteine sulfoxide (4S-β-CECO)

	U^{11}	U^{22}	U^{33}	U^{12}	U^{13}	U^{23}
S1	0.0326 (4)	0.0258 (3)	0.0231 (3)	0.0006 (3)	0.0103 (2)	0.0007 (3)
O3	0.0445 (12)	0.0420 (12)	0.0518 (12)	-0.0018 (12)	0.0314 (10)	0.0005 (13)
C6	0.0311 (15)	0.0214 (14)	0.0370 (15)	-0.0016 (11)	0.0177 (13)	-0.0009 (12)
O5	0.0316 (11)	0.0408 (13)	0.0408 (12)	0.0005 (10)	0.0131 (10)	-0.0147 (10)
C3	0.0342 (14)	0.0269 (15)	0.0218 (12)	-0.0016 (11)	0.0118 (11)	-0.0013 (11)
C4	0.0340 (15)	0.0250 (14)	0.0357 (16)	0.0050 (11)	0.0102 (13)	-0.0008 (12)
O4	0.0291 (12)	0.0480 (15)	0.076 (2)	-0.0046 (10)	0.0218 (13)	-0.0300 (14)
C2	0.0158 (11)	0.0262 (13)	0.0181 (11)	-0.0002 (9)	0.0050 (9)	0.0005 (10)
C5	0.0296 (14)	0.0266 (13)	0.0412 (17)	-0.0007 (11)	0.0139 (13)	-0.0082 (12)
O2	0.0300 (11)	0.0500 (14)	0.0287 (10)	-0.0168 (10)	0.0025 (9)	0.0072 (10)
O1	0.0285 (10)	0.0407 (12)	0.0189 (10)	-0.0044 (8)	0.0050 (8)	0.0003 (8)
N1	0.0210 (11)	0.0299 (12)	0.0188 (11)	0.0004 (9)	0.0018 (9)	0.0014 (9)
C1	0.0187 (12)	0.0261 (13)	0.0197 (12)	0.0002 (9)	0.0044 (10)	-0.0018 (10)

Table S12. Geometric parameters (Å, º) for (4S)-S-(2-carboxyethyl)-L-cysteine sulfoxide (4S-β-CECO)

S1—O3	1.498 (2)	C3—C2	1.529 (4)
S1—C3	1.811 (3)	C4—C5	1.511 (4)
S1—C4	1.812 (3)	C2—N1	1.485 (3)
C6—O5	1.319 (4)	C2—C1	1.529 (3)
C6—O4	1.200 (4)	O2—C1	1.238 (4)
C6—C5	1.506 (4)	O1—C1	1.253 (3)
O5—H5	0.71 (5)	N1—H1A	0.88 (3)

N1—H1B	0.89 (4)	N1—H1C	0.89 (3)
C2—H2	0.98	C3—H3A	0.97
C3—H3B	0.97	C4—H4A	0.97
C4—H4B	0.97	C5—H5A	0.97
C5—H5B	0.97		
O3—S1—C3	106.19 (15)	C3—C2—C1	111.3 (2)
O3—S1—C4	108.58 (15)	N1—C2—C3	107.2 (2)
C3—S1—C4	96.56 (15)	N1—C2—C1	110.6 (2)
O5—C6—C5	113.2 (3)	C6—C5—C4	110.7 (3)
O4—C6—O5	122.9 (3)	O2—C1—C2	119.1 (2)
O4—C6—C5	123.9 (3)	O2—C1—O1	125.3 (3)
C2—C3—S1	112.44 (19)	O1—C1—C2	115.5 (2)
C5—C4—S1	115.8 (2)	C6—O5—H5	108 (4)
C2—N1—H1A	106 (2)	C2—N1—H1B	113 (2)
C2—N1—H1C	115 (2)	H1A—N1—H1B	112 (4)
H1A—N1—H1C	104 (4)	H1B—N1—H1C	107 (3)
N1—C2—H2	109	C1—C2—H2	109
C3—C2—H2	109	S1—C3—H3A	109
S1—C3—H3B	109	C2—C3—H3A	109
C2—C3—H3B	109	H3A—C3—H3B	108
S1—C4—H4A	108	S1—C4—H4B	108
C5—C4—H4A	108	C5—C4—H4B	108
H4A—C4—H4B	107	C4—C5—H5A	109
C4—C5—H5B	110	C6—C5—H5A	110
C6—C5—H5B	110	H5A—C5—H5B	108
O3—S1—C3—C2	66.1 (3)	C4—S1—C3—C2	177.6 (2)
O3—S1—C4—C5	38.7 (3)	C3—S1—C4—C5	-70.8 (3)
O1—C1—C2—N1	167.4 (2)	O1—C1—C2—C3	-73.6 (3)
O2—C1—C2—N1	-15.2 (4)	O2—C1—C2—C3	103.8 (3)
N1—C2—C3—S1	-165.9 (2)	C1—C2—C3—S1	73.1 (3)
S1—C4—C5—C6	164.4 (2)	C4—C5—C6—O4	-10.8 (5)
C4—C5—C6—O5	170.3 (3)	O3—S1—C3—H3A	-173
O3—S1—C3—H3B	-55	C4—S1—C3—H3A	-61
C4—S1—C3—H3B	56	O3—S1—C4—H4A	161
O3—S1—C4—H4B	-83	C3—S1—C4—H4A	51

C3—S1—C4—H4B	167	H5—O5—C6—O4	-3 (5)
H5—O5—C6—C5	176 (5)	H1A—N1—C2—C1	-43 (3)
H1A—N1—C2—C3	-165 (3)	H1A—N1—C2—H2	77
H1B—N1—C2—C1	79 (3)	H1B—N1—C2—C3	-42 (3)
H1B—N1—C2—H2	-161	H1C—N1—C2—C1	-158 (3)
H1C—N1—C2—C3	80 (3)	H1C—N1—C2—H2	-38
O1—C1—C2—H2	47	O2—C1—C2—H2	-135
N1—C2—C3—H3A	73	N1—C2—C3—H3B	-45
C1—C2—C3—H3A	-48	C1—C2—C3—H3B	-166
H2—C2—C3—S1	-48	H2—C2—C3—H3A	-169
H2—C2—C3—H3B	74	S1—C4—C5—H5A	-75
S1—C4—C5—H5B	44	H4A—C4—C5—C6	42
H4A—C4—C5—H5A	163	H4A—C4—C5—H5B	-78
H4B—C4—C5—C6	-74	H4B—C4—C5—H5A	47
H4B—C4—C5—H5B	165	H5A—C5—C6—O4	-132
H5A—C5—C6—O5	49	H5B—C5—C6—O4	110
H5B—C5—C6—O5	-69		

Table S13. Hydrogen-bond geometry (Å, º) for (4S)-S-(2-carboxyethyl)-L-cysteine sulfoxide (4S-β-CECO)

D—H⋯*A*	*D*—H	H⋯*A*	*D*⋯*A*	*D*—H⋯*A*
O5—H5⋯O2^{i}	0.71(5)	1.94(5)	2.644(4)	172(6)
N1—H1*A*⋯O4^{ii}	0.88(3)	2.16(4)	2.847(4)	135(4)
N1—H1*A*⋯O5^{iii}	0.88(3)	2.51(4)	3.099(4)	125(4)
N1—H1*B*⋯O1^{iv}	0.89(4)	1.94(4)	2.822(4)	171(3)
N1—H1*C*⋯O1^{v}	0.89(3)	1.97(3)	2.818(4)	158(3)
N1—H1*C*⋯O2^{v}	0.89(3)	2.33(3)	3.077(3)	141(3)
C2—H2⋯O2^{vi}	0.98	2.51	3.421(4)	155
C3—H3*A*⋯O3^{vii}	0.97	2.56	3.314(4)	135
C5—H5*A* ⋯O4^{vi}	0.97	2.58	3.392(5)	142

Symmetry codes: (i) 2-x,-1/2+y,1-z; (ii) 2-x,1/2+y,1-z; (iii) 1-x,1/2+y,1-z; (iv) x,y,-1+z; (v) -1+x,y,-1+z; (vi) -1+x,y,z; (vii) 1+x,y,z.

Table S14. Viability and transcriptional factor induction fold in NRK-52E derived reporter cells treated with specific signaling pathway inducers and inhibitors for 18 hours. For viability, N=21; for specific TFs, N=3.

	GFP fluorescence		**NF-κB**		**Nrf2**		**MTF-1**		**p53**		**HSF-1**		**ATF2/3/4**		**ATF6**	
Treatment	Fold	SD	Fold	SD	Fold	SD	Fold	SD	Fold	SD	Fold	SD	Fold	SD	Fold	SD
Control	1.00	0.04	1.00	0.09	1.00	0.05	1.00	0.02	1.00	0.07	1.00	0.04	1.00	0.04	1.00	0.08
200 ng/mL LPS	0.95	0.05	8.44	0.44	1.00	0.13	1.10	0.07	1.05	0.03	0.58	0.03	1.28	0.13	0.74	0.08
10 ng/mL IL-1β	0.94	0.05	6.40	0.25	0.87	0.08	0.93	0.04	0.97	0.02	0.57	0.02	1.27	0.14	0.51	0.01
10 ng/mL TNF-α	0.90	0.03	5.67	0.23	1.01	0.05	0.72	0.05	1.05	0.03	0.70	0.05	1.37	0.21	0.76	0.04
500 nM CDDO-Me	0.77	0.05	0.72	0.12	75.4	4.3	1.93	0.07	0.46	0.01	0.45	0.05	6.38	0.64	4.34	0.46
25 µM Nutlin	0.61	0.05	0.53	0.04	1.27	0.09	0.46	0.02	2.41	0.06	0.51	0.04	4.38	0.17	0.14	0.03
20 µM tBHQ	0.98	0.05	0.82	0.07	37.2	3.9	1.73	0.05	1.70	0.09	1.35	0.06	4.95	0.17	1.26	0.25
10 µM CdCl2	1.03	0.04	1.21	0.09	108.2	5.9	3.90	0.07	1.32	0.11	4.53	0.54	2.54	0.23	2.56	0.37
100 µM ZnSO4	0.98	0.14	2.08	0.17	3.81	0.84	6.33	0.49	1.39	0.04	0.67	0.06	1.56	0.27	0.85	0.09
250 µM CoCl2	0.84	0.05	0.54	0.12	22.8	4.3	1.75	0.12	1.47	0.40	1.29	0.20	2.88	0.26	1.14	0.12
50 nM Thapsigargin	0.86	0.05	1.06	0.20	0.76	0.06	0.44	0.01	0.24	0.01	0.24	0.02	5.20	0.21	28.6	1.8
240 µM H_2O_2	0.81	0.06	0.68	0.06	1.36	0.11	0.79	0.12	0.76	0.02	0.75	0.09	2.89	0.07	0.61	0.12
250 nM 17-AAG	0.72	0.05	0.74	0.06	1.24	0.08	2.15	0.18	1.97	0.07	0.64	0.06	2.12	0.16	0.94	0.11
80 µM pyocyanin	0.84	0.04	1.61	0.05	222.5	8.5	1.36	0.05	2.26	0.07	1.46	0.05	2.57	0.27	1.31	0.18

Table S15. Viability and transcriptional factor induction fold in NRK-52E derived reporter cells treated with β-CEC, CMC, and their sulfoxides for 18 hours. For viability, N=21; for specific TFs, N=3.

		GFP fluorescence		**NF-κB**		**Nrf2**		**MTF-1**		**p53**		**HSF-1**		**ATF2/3/4**		**ATF6**	
Treatment	conc, mM	Fold	SD	Fold	SD	Fold	SD	Fold	SD	Fold	SD	Fold	SD	Fold	SD	Fold	SD
β-CEC	0.5	1.084	0.070	0.828	0.093	1.003	0.034	1.170	0.049	0.986	0.117	0.851	0.040	1.259	0.146	0.839	0.172
	1	1.030	0.063	0.971	0.112	1.100	0.124	1.186	0.039	1.047	0.060	0.940	0.100	1.680	0.054	0.905	0.131
	2	1.029	0.046	1.059	0.199	1.275	0.131	1.158	0.040	0.996	0.042	0.923	0.059	2.370	0.092	0.797	0.091
	4	1.028	0.041	1.037	0.217	1.871	0.126	1.144	0.080	1.085	0.072	1.036	0.100	3.287	0.137	0.717	0.041
R-β-CECO	0.5	1.053	0.078	0.762	0.100	0.871	0.026	1.196	0.027	1.054	0.076	0.890	0.062	0.828	0.013	0.751	0.178
	1	1.016	0.097	0.737	0.035	0.817	0.096	1.151	0.049	1.043	0.024	0.935	0.156	0.792	0.009	0.984	0.228
	2	0.988	0.048	0.837	0.071	0.912	0.073	1.163	0.038	1.026	0.028	0.915	0.085	0.873	0.075	1.144	0.114
	4	1.078	0.075	0.784	0.048	0.901	0.044	1.003	0.027	1.061	0.075	1.057	0.131	0.872	0.058	1.026	0.089
S-β-CECO	0.5	1.039	0.027	0.693	0.122	0.978	0.075	1.218	0.046	1.000	0.058	0.789	0.101	0.884	0.037	0.697	0.061
	1	1.023	0.045	0.669	0.115	0.997	0.094	1.200	0.024	0.992	0.049	0.798	0.156	0.891	0.100	0.763	0.073
	2	0.993	0.069	0.810	0.047	1.114	0.077	1.229	0.018	1.014	0.029	0.915	0.100	0.975	0.060	0.900	0.115
	4	1.070	0.066	0.839	0.058	1.712	0.066	1.155	0.012	1.103	0.014	1.092	0.182	0.910	0.014	0.818	0.082
CMC	0.5	1.032	0.065	0.836	0.043	1.080	0.107	0.991	0.062	0.922	0.035	0.803	0.062	1.376	0.041	0.807	0.131
	1	1.000	0.050	0.744	0.019	1.340	0.141	1.035	0.048	0.854	0.127	0.702	0.079	2.181	0.351	0.748	0.064
	2	0.975	0.043	0.998	0.359	1.869	0.064	1.139	0.063	0.973	0.099	0.762	0.094	3.733	0.336	0.655	0.141
	4	1.006	0.039	0.926	0.110	2.712	0.136	1.224	0.025	0.925	0.057	0.755	0.071	4.711	0.376	0.583	0.058
R-CMCO	0.5	0.985	0.082	0.821	0.025	1.104	0.074	1.030	0.044	1.003	0.062	0.782	0.022	1.084	0.032	0.611	0.070
	1	0.945	0.072	0.768	0.023	1.373	0.054	0.981	0.075	0.984	0.029	0.671	0.051	1.549	0.145	0.752	0.203
	2	0.938	0.034	0.823	0.080	2.269	0.240	1.076	0.055	1.062	0.039	0.765	0.087	2.698	0.280	0.702	0.054
	4	1.031	0.062	0.843	0.036	4.670	0.262	0.972	0.068	1.108	0.069	0.780	0.122	3.491	0.476	0.567	0.084
S-CMCO	0.5	1.007	0.066	0.802	0.120	1.075	0.137	0.992	0.033	1.043	0.009	0.704	0.064	1.108	0.021	0.584	0.052
	1	0.990	0.070	0.750	0.047	1.070	0.114	1.069	0.042	1.042	0.060	0.770	0.098	0.930	0.060	0.629	0.085
	2	0.983	0.081	0.809	0.123	1.158	0.086	1.052	0.018	1.128	0.023	0.929	0.127	1.058	0.055	0.816	0.131
	4	1.027	0.058	0.758	0.088	1.598	0.145	1.012	0.029	1.250	0.127	0.923	0.013	1.145	0.071	0.620	0.116

Table S16. Viability and activation of the amino acid stress response in NRK-52E cells treated with β-CEC, CMC, and a panel of L-amino acids.

Treatment →		4 mM β-CEC				4 mM CMC			
		GFP fluorescence		Luciferase luminesc		GFP fluorescence		Luciferase luminesc	
Co-treatment	conc, mM	Fold	SD	Fold	SD	Fold	SD	Fold	SD
none	-	1.026	0.124	3.283	0.261	0.996	0.044	5.741	0.258
alanine	1	0.982	0.032	3.879	0.262	1.024	0.025	6.020	0.373
	2	1.068	0.007	3.956	0.284	1.082	0.029	5.883	0.581
	4	1.054	0.035	3.705	0.105	1.078	0.020	6.226	0.293
valine	0.5	1.033	0.034	3.242	0.213	1.133	0.064	5.390	0.703
	1	0.993	0.052	3.211	0.254	1.110	0.029	5.406	0.068
	2	1.006	0.023	3.627	0.177	1.125	0.016	5.789	0.299
	4	0.991	0.037	3.806	0.202	1.090	0.036	5.529	0.270
leucine	0.5	1.026	0.058	3.020	0.159	1.007	0.066	5.753	0.528
	1	1.033	0.093	2.928	0.301	1.128	0.034	5.162	0.143
	2	1.064	0.017	3.666	0.201	1.115	0.076	5.970	0.441
	4	1.058	0.077	3.711	0.285	1.152	0.020	5.719	0.272
isoleucine	1	1.011	0.033	3.534	0.253	1.092	0.013	5.712	0.343
	2	1.016	0.035	3.818	0.382	1.049	0.018	5.883	0.231
	4	0.976	0.089	4.211	0.189	1.044	0.069	6.113	0.361
methionine	0.5	1.055	0.077	3.413	0.433	1.104	0.005	5.630	0.143
	1	0.986	0.102	3.348	0.258	1.029	0.065	5.206	0.825
	2	0.998	0.074	3.103	0.289	1.027	0.007	5.181	0.102
	4	1.024	0.096	2.562	0.204	1.023	0.044	3.753	0.369
phenylalanine	0.5	1.020	0.040	3.760	0.127	0.981	0.028	6.170	0.207
	1	1.018	0.015	3.644	0.077	1.008	0.047	6.599	0.225
	2	0.985	0.036	3.999	0.131	0.990	0.018	6.496	0.233
	4	1.005	0.018	3.876	0.344	0.964	0.125	6.077	0.167
threonine	0.5	1.052	0.028	3.293	0.150	1.072	0.059	5.986	0.540
	1	1.034	0.045	3.890	0.483	1.065	0.170	6.174	0.419
	2	1.089	0.048	4.340	0.347	0.984	0.053	6.958	0.248
	4	0.998	0.043	4.679	0.161	1.006	0.082	6.346	0.618
lysine	1	0.979	0.071	3.474	0.059	0.993	0.063	5.640	0.532
	2	1.040	0.058	3.348	0.143	1.002	0.070	5.682	0.456
	4	1.015	0.012	3.263	0.268	1.009	0.105	5.743	0.603
glutamic acid	1	1.041	0.025	4.086	0.273	1.005	0.023	6.404	0.412
	2	1.108	0.051	5.095	0.153	1.048	0.023	6.157	0.594
	4	1.027	0.035	5.713	0.254	0.993	0.031	6.770	0.406
histidine	0.5	1.056	0.059	3.846	0.312	1.063	0.015	6.082	0.114
	1	1.021	0.057	3.660	0.222	1.067	0.098	5.941	0.578
	2	1.065	0.043	3.392	0.151	1.061	0.027	5.753	0.379
	4	0.974	0.033	2.486	0.152	1.034	0.052	4.656	0.347
tryptophan	0.5	1.037	0.089	3.560	0.186	1.059	0.119	5.311	0.708
	1	0.995	0.021	3.309	0.055	1.032	0.072	4.787	0.247
	2	1.040	0.030	2.952	0.116	1.008	0.061	4.351	0.346
	4	1.002	0.019	2.304	0.049	1.035	0.101	3.358	0.287
N-acetylcysteine	1	0.984	0.036	2.467	0.028	0.962	0.033	4.217	0.435
	2	1.007	0.012	0.858	0.054	0.981	0.007	0.843	0.045
	4	0.893	0.032	1.097	0.070	0.860	0.034	1.139	0.070

Table S17. Viability and activation of the DNA stress response in NRK-52E cells treated with a panel of anthracycline drugs, other nephrotoxic drugs and β-CEC.

Co-treatment →		None				1 mM β-CEC			
		GFP fluorescence		Luciferase luminesc		GFP fluorescence		Luciferase luminesc	
Treatment	conc, nM	Fold	SD	Fold	SD	Fold	SD	Fold	SD
Daunorubicin	62.5	0.94	0.07	2.54	0.12	0.93	0.06	2.39	0.11
	125	0.71	0.07	4.33	0.06	0.65	0.05	4.44	0.10
	250	0.56	0.05	6.91	0.04	0.52	0.03	7.06	0.09
	500	0.59	0.04	5.69	0.07	0.65	0.07	4.59	0.08
Doxorubicin	62.5	1.10	0.03	3.44	0.06	1.05	0.03	3.36	0.05
	125	0.84	0.01	5.86	0.05	0.85	0.04	5.69	0.04
	250	0.59	0.03	3.89	0.03	0.67	0.04	3.63	0.05
	500	0.54	0.09	1.55	0.01	0.63	0.03	1.34	0.06
Epirubicin	62.5	1.08	0.05	1.60	0.05	1.19	0.02	1.45	0.11
	125	1.11	0.06	2.76	0.08	1.04	0.03	3.01	0.44
	250	0.73	0.02	4.22	0.10	0.75	0.02	4.38	0.13
	500	0.63	0.03	3.68	0.18	0.56	0.02	4.18	0.24
Idarubicin	62.5	0.94	0.06	3.07	0.08	1.00	0.03	2.58	0.04
	125	0.83	0.03	5.39	0.02	0.79	0.13	5.70	0.06
	250	0.57	0.02	7.14	0.03	0.57	0.04	7.52	0.06
	500	0.49	0.03	10.59	0.07	0.51	0.06	10.20	0.27
Mitoxantrone	125	1.10	0.05	1.21	0.16	1.05	0.04	1.32	0.15
	250	0.95	0.05	1.77	0.17	0.90	0.08	1.79	0.05
	500	0.82	0.03	2.30	0.17	0.71	0.05	2.43	0.09
	1000	0.70	0.09	3.48	0.14	0.65	0.02	3.55	0.14
	conc, mM								
Gentamicin	0.5	1.16	0.02	1.22	0.05	1.10	0.05	1.34	0.12
	1	1.14	0.05	1.52	0.13	1.16	0.02	1.55	0.05
	2	1.18	0.00	2.09	0.19	1.08	0.02	2.18	0.12
	4	0.96	0.04	3.49	0.28	0.94	0.02	3.39	0.03
	8	0.56	0.06	0.02	0.01	0.54	0.03	0.01	0.00
	conc, µM								
Carboplatin	16	1.13	0.09	1.56	0.09	1.18	0.05	1.53	0.01
	32	1.17	0.07	1.78	0.14	1.18	0.03	1.85	0.02
	64	1.12	0.04	2.36	0.05	1.09	0.05	2.23	0.06
	128	1.04	0.02	2.87	0.05	1.01	0.05	2.73	0.12
Mitomycin	0.25	1.15	0.01	2.06	0.10	1.08	0.02	2.36	0.01
	0.5	1.02	0.03	2.63	0.05	1.04	0.04	2.79	0.01
	1	1.05	0.03	3.83	0.04	1.05	0.07	4.14	0.08
	2	1.02	0.04	5.53	0.14	1.00	0.12	5.61	0.06

Table S18. Viability of NRK-52E cells treated with a panel of environmental nephrotoxic pollutants and β-CEC.

Co-treatment →		None		1 mM β-CEC	
Treatment	conc, μM	GFP Fold	SD	GFP Fold	SD
$NaAsO_2$	3.8	0.95	0.04	1.04	0.03
	7.5	1.11	0.03	0.71	0.06
	15	0.95	0.07	0.56	0.11
	30	0.44	0.02	0.23	0.02
$CdCl_2$	7.5	1.02	0.06	1.20	0.13
	15	1.21	0.03	1.04	0.07
	30	1.10	0.04	0.33	0.02
	60	0.55	0.02	0.27	0.04
$CuCl_2$	120	1.04	0.05	0.98	0.06
	240	0.86	0.06	0.94	0.12
	480	0.35	0.08	0.70	0.05
	960	0.12	0.03	0.26	0.07
$HgCl_2$	7.5	1.08	0.02	1.06	0.03
	15	1.16	0.07	0.86	0.05
	30	1.15	0.04	0.25	0.09
	60	0.53	0.05	0.13	0.02
$Pb(OAc)_2$	150	1.00	0.05	1.01	0.04
	300	1.04	0.02	0.95	0.04
	600	0.99	0.06	0.92	0.07
	1200	0.89	0.09	0.53	0.07
Atrazin	150	0.90	0.07	0.90	0.01
	300	0.84	0.07	0.89	0.06
	600	0.76	0.02	0.82	0.06
	1200	0.65	0.06	0.69	0.03
2,4-D	150	0.94	0.03	1.07	0.14
	300	1.03	0.03	1.08	0.08
	600	0.94	0.02	1.02	0.06
	1200	0.95	0.01	1.06	0.07
Diquat	75	1.07	0.02	1.04	0.02
	150	1.20	0.02	1.01	0.03
	300	0.28	0.04	0.36	0.06
	600	0.16	0.02	0.18	0.03
Paraquat	75	1.01	0.07	0.98	0.04
	150	1.05	0.09	0.98	0.04
	300	1.12	0.05	1.11	0.07
	600	1.09	0.09	1.05	0.05
Ochratoxin A	7.5	1.00	0.02	0.89	0.06
	15	0.80	0.02	0.79	0.06
	30	0.50	0.04	0.58	0.02
	60	0.41	0.04	0.44	0.02

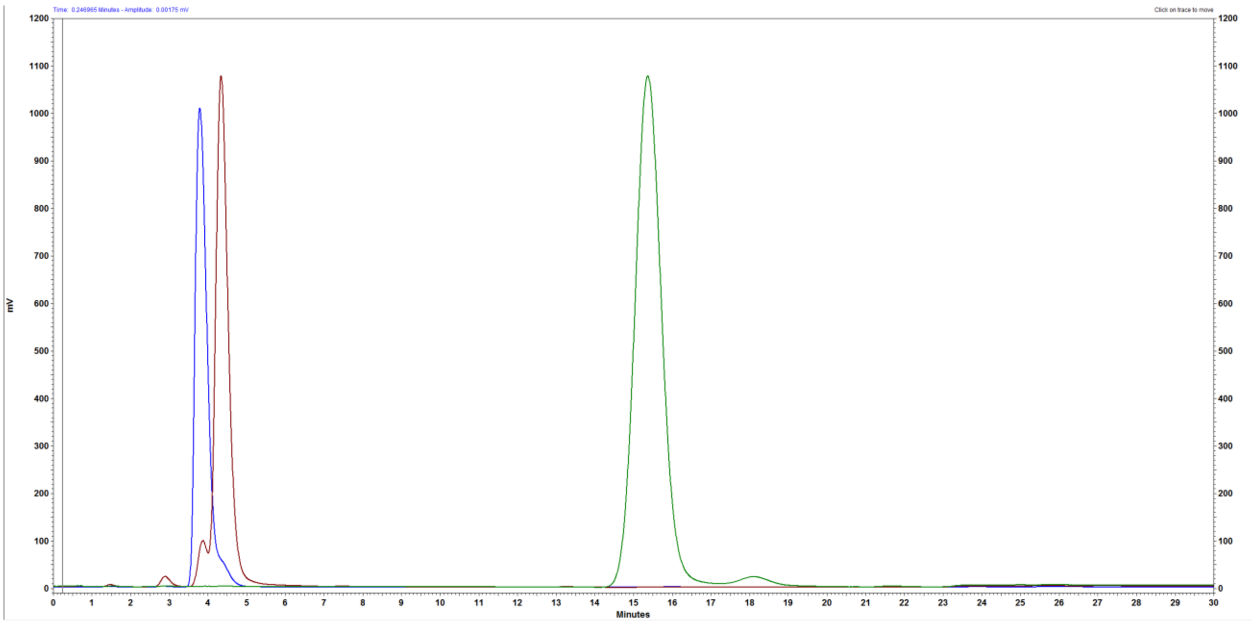

Figure S1. Ion-exchange chromatograms of *S*-(2-carboxyethyl)-L-cysteine (β-CEC, green at 15.37 min), (2*R*,4*R*)-*S*-(2-carboxyethyl)-cysteine sulfoxide (4*R*-β-CECO, blue at 3.79 min), and (2*R*,4*S*)-*S*-(2-carboxyethyl)-cysteine sulfoxide (4*S*-β-CECO, red at 4.34 min).

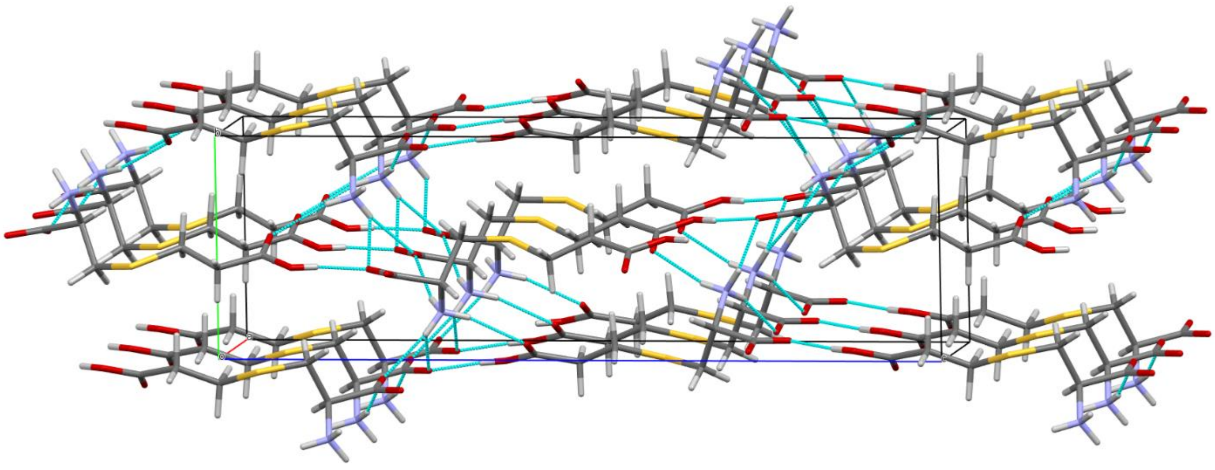

Figure S2. The molecular packing in *S*-(2-carboxyethyl)-L-cysteine (β-CEC, **1**). A view of the unit-cell contents shown in projection down the ***a*** axis. Color code for crystallographic axes: red - ***a***, green - ***b***, blue - ***c***. Hydrogen bonds are shown as cyan dotted lines.

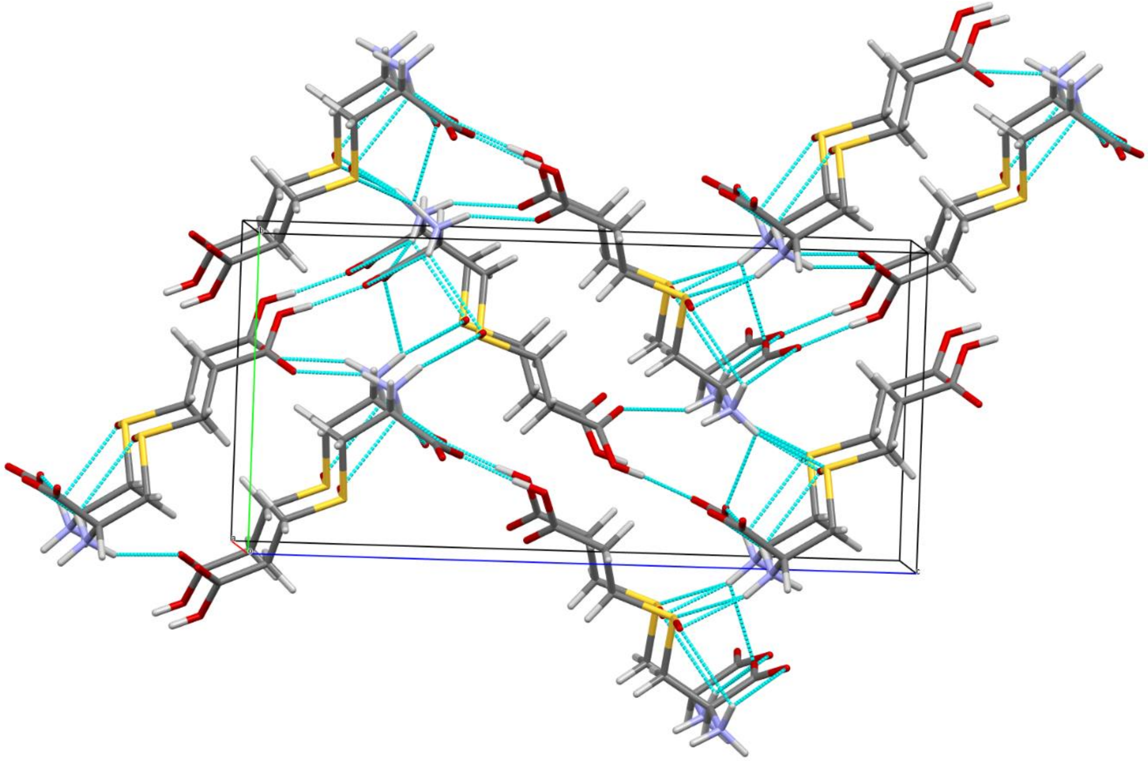

Figure S3. The molecular packing in (2*R*,4*R*)-*S*-(2-carboxyethyl)-cysteine sulfoxide [(4*R*)-β-CECO, **2**]. A view of the unit-cell contents shown in projection down the ***a*** axis. Color code for crystallographic axes: red - ***a***, green - ***b***, blue - ***c***. Hydrogen bonds are shown as cyan dotted lines.

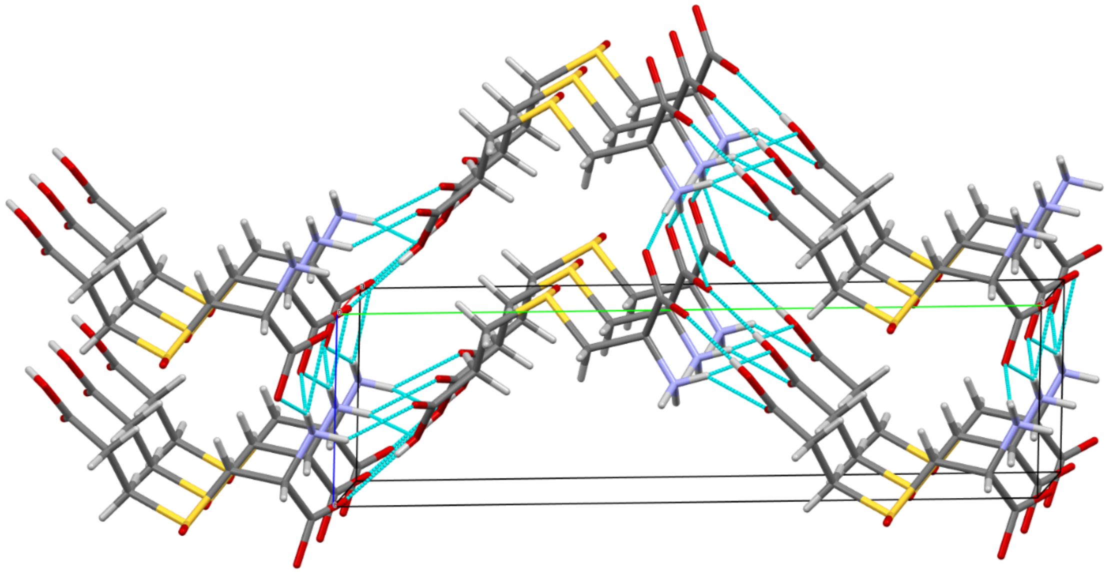

Figure S4. The molecular packing in (2*R*,4*S*)-*S*-(2-carboxyethyl)-cysteine sulfoxide [(4*S*)-β-CECO, **3**]. A view of the unit-cell contents shown in projection down the ***a*** axis. Color code for crystallographic axes: red - ***a***, green - ***b***, blue - ***c***. Hydrogen bonds are shown as cyan dotted lines.

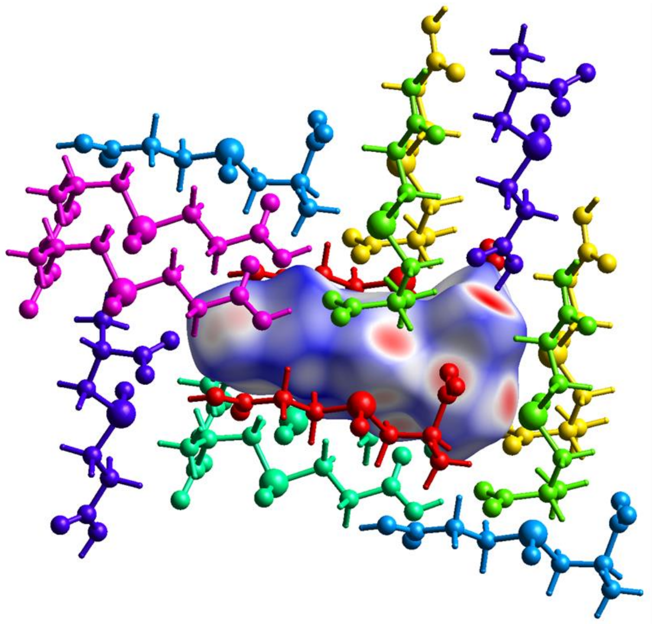

Pairwise interactions energies (kJ/mol)
B3LYP/6-31G(d,p) electron densities energy model

Color code	N	Symm operator	E elstat	E polar	E disp	E repuls	E total
	2	x, y, z	-3.6	-23.3	-25.3	42.7	-16.7
	2	-x, y+1/2, -z+1/2	-81.8	-26.5	-28.2	34.5	-109.3
	2	-x, y+1/2, -z+1/2	-68.4	-18.9	-16.9	35.7	-79
	2	x+1/2, -y+1/2, -z	-36.5	-16.8	-34.1	64.9	-40.7
	2	x, y, z	2.6	-2.1	-2.8	0	-1.2
	2	-x+1/2, -y, z+1/2	-96.9	-26.3	-8	116.3	-57.1
	2	x+1/2, -y+1/2, -z	-9	-1.6	-15.3	14.5	-15.1

Figure S5. Interaction energies in crystal structure of **2**. Left, a view of interactions between a central (4*R*)-β-CECO molecule, shown as its Hirshfeld surface, and 14 molecules that share the interaction surfaces with the central molecule. Right, calculated energies (electrostatic, polarization, dispersion, repulsion, and total) of pairwise interactions in **2** between the central molecule and those indicated by respective colors.

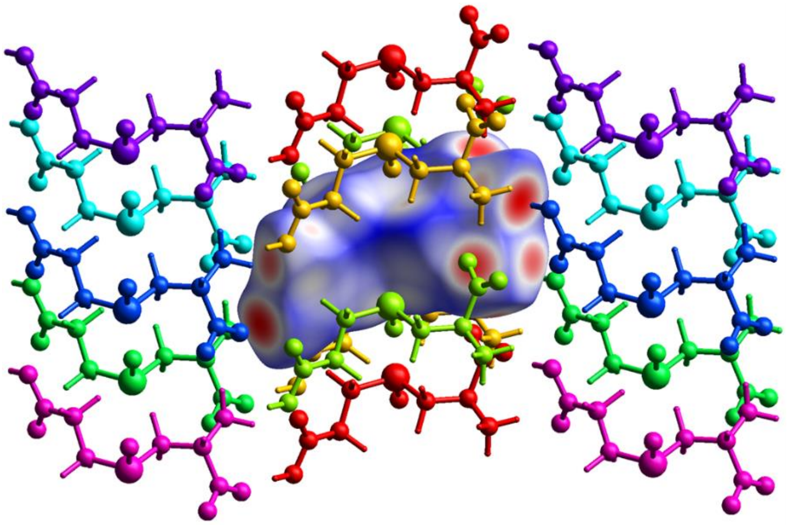

Pairwise interactions energies (kJ/mol)
B3LYP/6-31G(d,p) electron densities energy model

Color code	N	Symm operator	E elstat	E polar	E disp	E repuls	E total
(red)	2	x, y, z	-106.7	-31.6	-22	60	-118.2
(orange)	2	x, y, z	13.2	-17.8	-27.3	27.5	-5.9
(yellow-green)	2	x, y, z	-61.4	-32.2	-32.1	60.6	-79.2
(green)	2	-x, y+1/2, -z	-5.5	-7.4	-9.2	6.3	-15.5
(cyan)	2	-x, y+1/2, -z	-16.3	-6.9	-9.2	2.3	-29
(blue)	2	-x, y+1/2, -z	-112.4	-29.1	-12.8	90.7	-95.5
(violet)	2	-x, y+1/2, -z	2	-1.5	-1.4	0	-0.3
(magenta)	2	-x, y+1/2, -z	11.8	-1.4	-0.9	0	10.7

Figure S6. Interaction energies in crystal structure of **3**. Left, a view of interactions between a central (4*S*)-β-CECO molecule, shown as its Hirshfeld surface, and 16 molecules that share the interaction surfaces with the central molecule. Right, calculated energies (electrostatic, polarization, dispersion, repulsion, and total) of pairwise interactions in **3** between the central molecule and those indicated by respective colors.

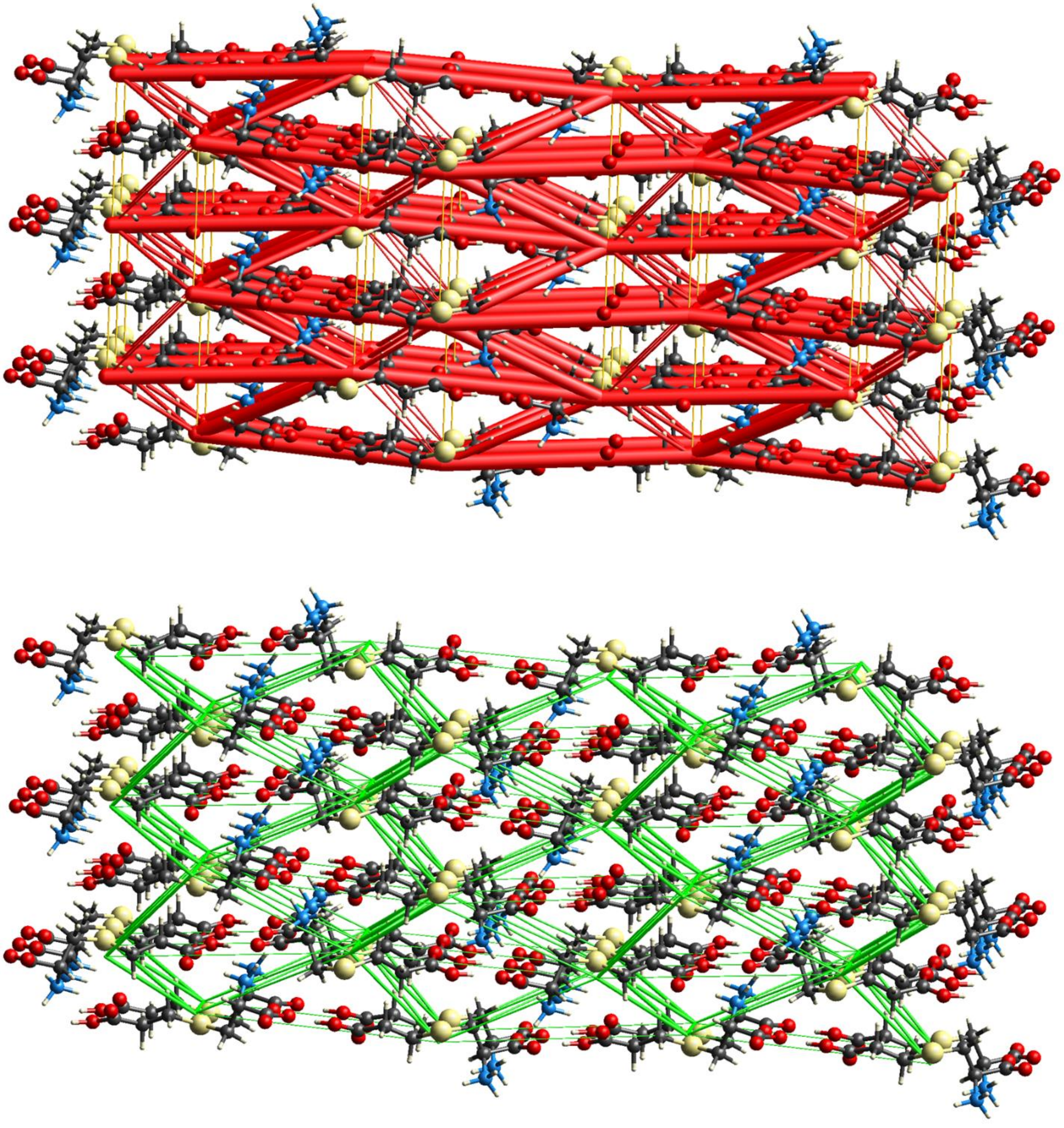

Figure S7. Energy framework for electrostatic (top) and dispersion (bottom) pairwise interaction energies in **1**. The cylinders link molecular centroids, and the cylinder thickness is proportional to the magnitude of the energies, such as those shown in the Figure 4(b). For clarity, the cylinders corresponding to energies <5 kJ mol^{-1} are not shown.

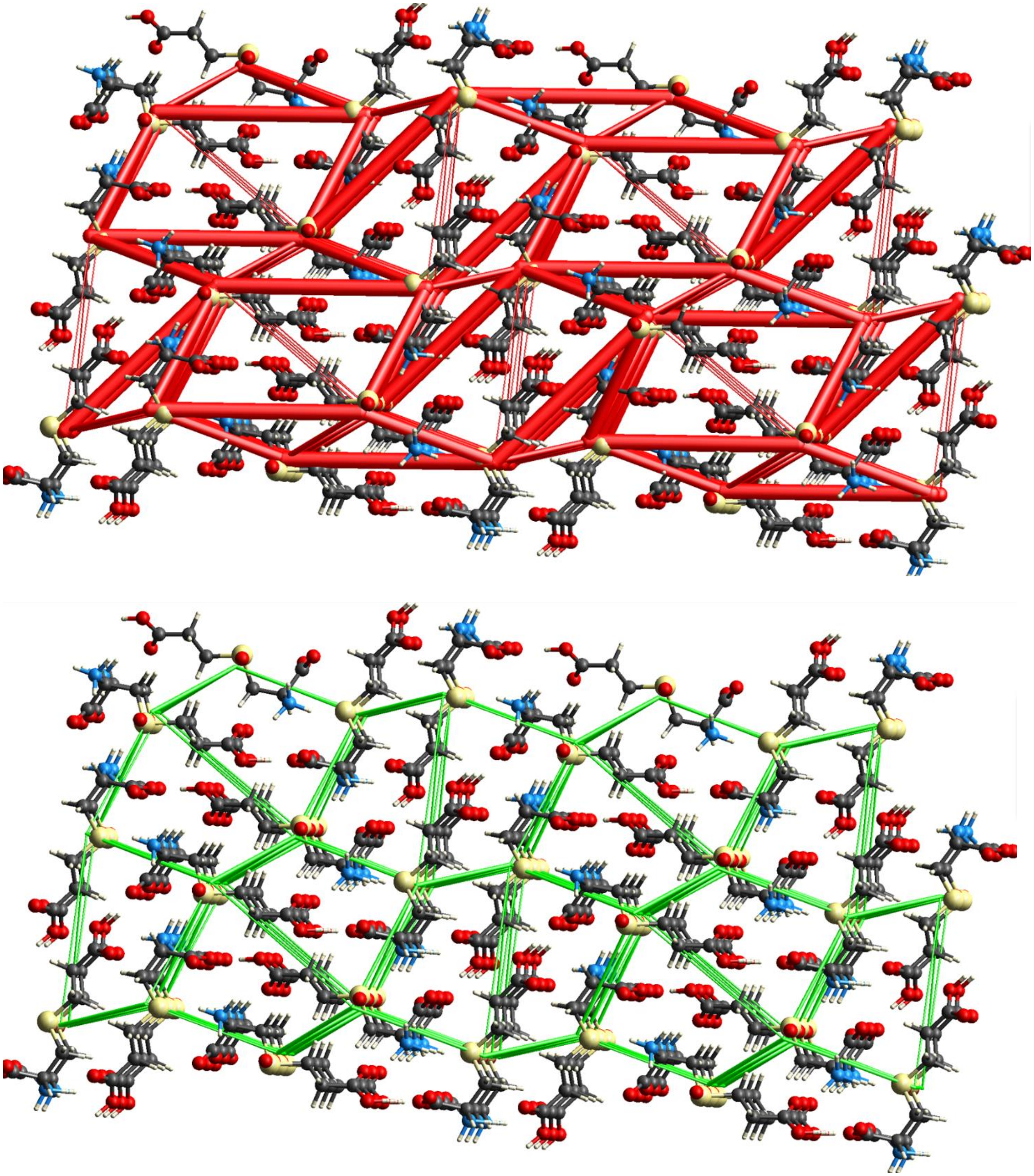

Figure S8. Energy framework for electrostatic (top) and dispersion (bottom) pairwise interaction energies in **2**. The cylinders link molecular centroids, and the cylinder thickness is proportional to the magnitude of the energies, such as those shown in the Supplementary Figure S5. For clarity, the cylinders corresponding to energies <5 kJ mol-1 are not shown.

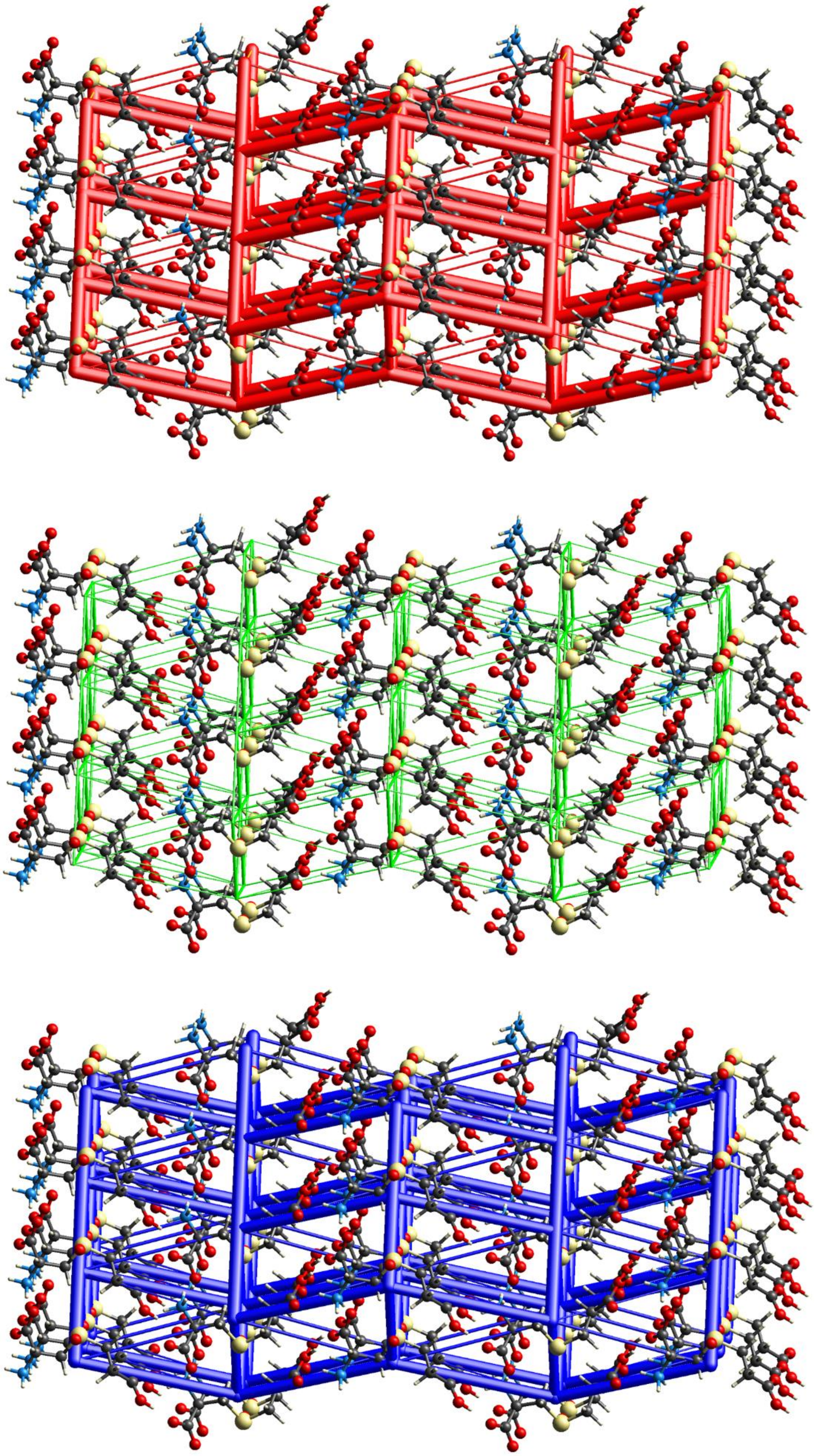

Figure S9. Energy framework for electrostatic (top), dispersion (middle), and total (bottom) pairwise interaction energies in **3**. The cylinders link molecular centroids, and the cylinder thickness is proportional to the magnitude of the energies, such as those shown in the Supplementary Figure S6. For clarity, the cylinders corresponding to energies <5 kJ mol-1 are not shown
